# Synthetic microbial communities: Sandbox and blueprint for soil health enhancement

**DOI:** 10.1002/imt2.172

**Published:** 2024-02-11

**Authors:** Mei Li, Jie Hu, Zhong Wei, Alexandre Jousset, Thomas Pommier, Xiangyang Yu, Yangchun Xu, Qirong Shen

**Affiliations:** ^1^ Jiangsu Key Laboratory for Food Quality and Safety—State Key Laboratory Cultivation Base of Ministry of Science and Technology, Institute of Food Safety and Nutrition Jiangsu Academy of Agricultural Sciences Nanjing China; ^2^ Department of Microbial Ecology Netherlands Institute of Ecology Wageningen The Netherlands; ^3^ Jiangsu Provincial Key Lab for Solid Organic Waste Utilization, Key Lab of Organic‐Based Fertilizers of China, Jiangsu Collaborative Innovation Center for Solid Organic Wastes, Educational Ministry Engineering Center of Resource‐Saving Fertilizers Nanjing Agricultural University Nanjing China; ^4^ UMR INRAE 1418 Ecologie Microbienne Université Claude Bernard Lyon 1 Villeurbanne France

## Abstract

We summarize here the use of SynComs in improving various dimensions of soil health, including fertility, pollutant removal, soil‐borne disease suppression, and soil resilience; as well as a set of useful guidelines to assess and understand the principles for designing SynComs to enhance soil health. Finally, we discuss the next stages of SynComs applications, including highly diverse and multikingdom SynComs targeting several functions simultaneously.

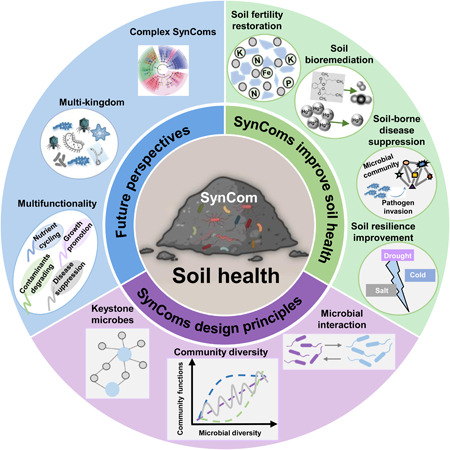

In nature, diverse microorganisms do not act as individuals but rather interact and communicate with one another in a dynamically changing microbial community, they are essential for maintaining the Earth's biosphere and for the survival of plants and animals as they contribute to nearly all biogeochemical cycles on earth. However, studying microbial populations directly in their natural environment poses significant challenges due to their vast population size and complex interaction network. This difficulty hampers our ability to predict the behavior of microbial communities in nature, thereby limiting our understanding of the functionality of microorganisms in the natural environmental systems and developing and utilizing microbial resources. Further, the inherent complexity of the natural microbiota makes it immensely challenging to establish causality and, subsequently, dissect mechanisms. One emerging strategy to tackle the aforementioned challenge is to use simplified, synthetic microbial communities (SynComs).

## SYNCOMS AND ITS APPLICATIONS IN SOIL HEALTH MANAGEMENT

**Figure 1:**
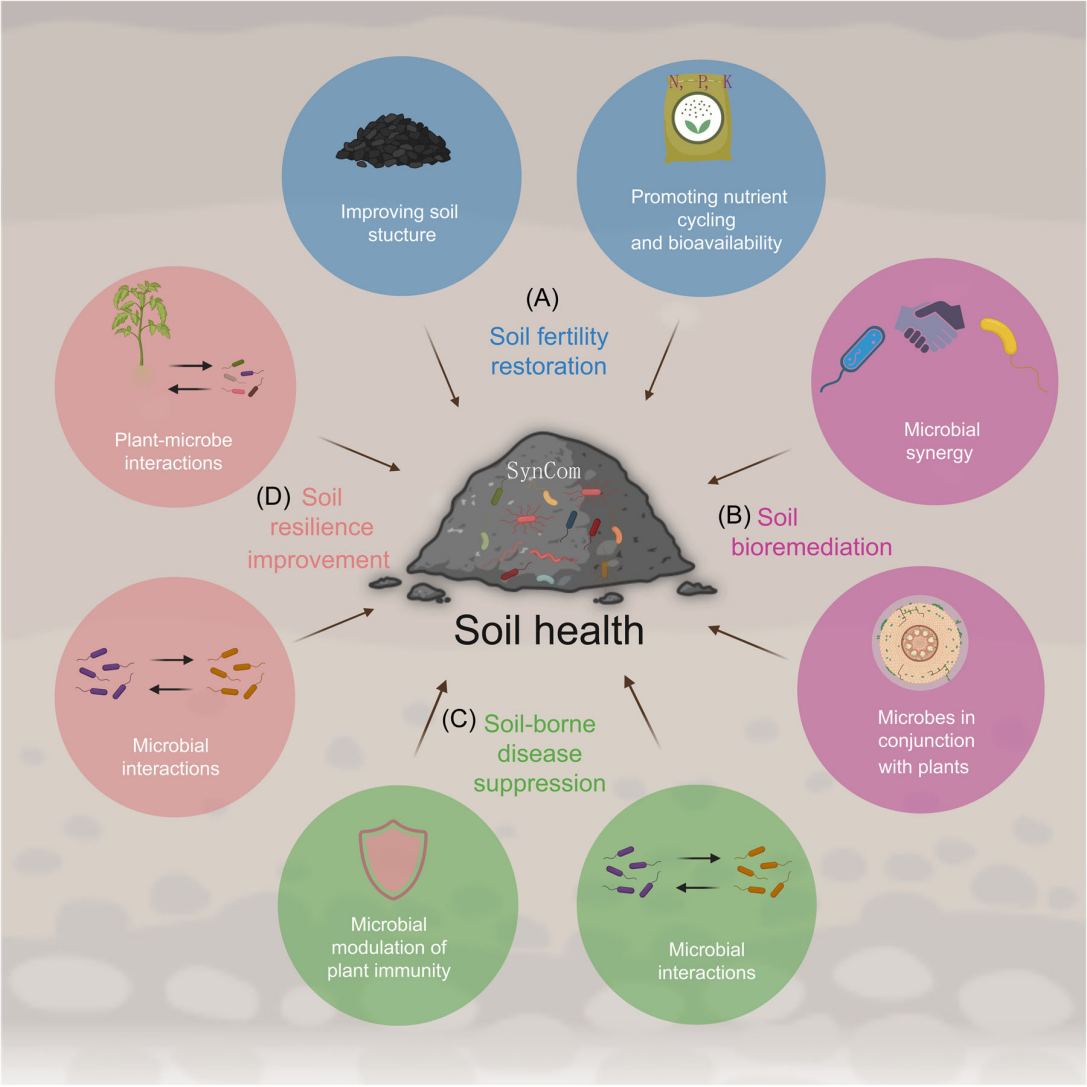
Synthetic microbial communities (SynComs) play essential roles in soil health improvement. SynComs applications for soil fertility restoration by improving soil structure or promoting nutrient cycling and bioavailability (A), for soil bioremediation through microbial synergy or by microbes in conjunction with plants (B), for soil‐borne disease suppression (C) and soil resilience improvement by microbial interactions or microbe‐plant interactions (D). The graphic was created with BioRender.com.

SynComs are artificial combinations of two or more distinct cultured microorganisms with well‐defined taxonomic status and specific functional characteristics. Compared with individual organisms, they are functionally redundant which implies they exhibit reduced metabolic burden due to a division of labor, exchange resources, possess expanded metabolic capabilities relative to monocultures, constantly communicate (chemically or physically), and hence better resist environmental perturbations or invasions by other species as such microbial communities [1]. Moreover, SynComs maintain key features of natural microbial communities and, because of their reduced complexity and defined nature, have been increasingly used as a model system to study functional, ecological, and structural concepts of native microbiota. Construction and application of SynComs have been demonstrated in different contexts, such as human health, marine bacteria–plankton interaction, and soil health. In the following sections, we review how SynComs have broad application potential in the field of soil health improvement, including soil fertility restoration, soil pollutant bioremediation, soil‐borne disease suppression, and soil resilience enhancement (Figure 1).

### SynComs applications for fertility restoration

Intensive agriculture has caused a variety of environmental issues such as exhaustion of soil organic matter and soil nutrient reserves, resulting in soil degradation. At present, one‐third of all global land surfaces are degraded to some extent, and 24 billion metric tons of fertile soil are lost every year [2]. Driven by a better understanding of the role of microorganisms in soil fertility and crop productivity, the applications of SynComs to restore soil fertility is increasingly studied. The applications of SynComs can restore soil fertility through improving soil structure, nutrient cycling, and bioavailability (Figure 1A).

Microorganisms are an important determinant of both aggregate formation and stabilization and this effect is consistent across soils. One of the main drivers of aggregate formation is the production of extracellular polymeric substances (EPS) by bacteria and fungi, which combine soil particles together [2]. The importance of synergetic interspecific interactions for EPS formation in soil bacteria suggests that SynComs maximizing EPS production may improve soil structure. Application of SynComs have revealed that biotic interactions are an essential determinant of soil aggregate formation. For instance, *Pseudomonas fluorescens* and *Stenotrophomonas maltophilia* isolated from *Agaricus lilaceps* fruiting body binds soil more than *Bacillus sp*. isolated from outside and inside of the fairy ring [3]. Additionally, fungal activity can alter the nature and extent of availability of pore spaces in the soil for the habitat of other surrounding microbes. Furthermore, a recent work studied the effects of trophic interactions on soil aggregation by building a simple synthetic community which included a protist and a bacteria/fungus. They found that the protist *Acanthamoeba castellanii* increased the formation of soil aggregates but decreased their stability, an effect the authors attributed to changes in the production of bacterial mucilage [4].

Soil microorganisms are involved in the cycle of several plant nutrients encountered in soil. Several microorganisms can for instance solubilize phosphorus (P) or microelements such as iron (Fe) or zinc (Zn) from the soil matrix, mineralize nitrogen (N) from the organic material pool and even fix nitrogen from the air. For instance, a study showed that N_2_‐fixing, P‐solubilizing, K‐solubilizing, and indole‐3‐acetic acid (IAA)‐producing bacteria can significantly increase the content of available N, P, and K in soil, and a combination of these growth‐promoting bacteria with different functions effectively improved plant N/P/K uptake and growth [5].

### SynComs applications for pollutant bioremediation

Soil pollution, such as pesticide residues, heavy metals, petroleum hydrocarbons, and microplastics, poses a serious threat to soil quality. Several microorganisms can naturally degrade pollutants, a property often implemented in bioremediation strategies. SynComs have been applied in various bioaugmentation strategies, with the goal of invading the soil microbiome with microorganisms harboring metabolic potential to degrade a specific contaminant (Figure 1B).

Given the high complexity of soil contaminants, bioaugmentation of single strains may not be sufficient to achieve a high “removal efficiency,” as demonstrated in the case of pesticide linuron [6]. *Variovorax sp*. strain WDL1 could degrade linuron via using it as carbon, nitrogen, and other energy sources directly. While *Delftia acidovorans* WDL34 and *Pseudomonas sp*. strain WDL5 were not able to directly use linuron, but they can use some intermediate of linuron's degradation. When strain WDL1 was mixed with the other bacteria which cannot use linuron as a source directly in a SynCom, they increased linuron degradation rate dramatically through synergetic interactions. Further, reductionist approaches with SynComs found that resident bacteria improved micropollutant 2,6‐Dichlorobenzamide degradation activity by cooperating with degrading bacteria *Aminobacter sp*. MSH1. This opens a door for assisting bioaugmentation through coinoculation with “helper” bacteria originating from and/or already adapted to the target environment [7].

Specific microbes such as plant growth‐promoting rhizobacteria (PGPR) have indirect positive impacts on the removal of contaminants by plants (phytoremediation). SynComs composed of PGPR could increase plant uptake and accumulation of contaminant by stimulating plant growth, which can be manipulated to improve the efficacy of phytoremediation. Additionally, inoculation of contaminated soils with SynComs composed of Arbuscular mycorrhiza and *Aspergillus terreus* at vegetation reintroduction not only enhanced the extraction of heavy metals from the polluted soils but also enabled the plants establishment on degraded soil and thereby improved soil health [8].

### SynComs applications for soil‐borne disease suppression

Plant diseases caused by soil‐borne pathogens are a major threat for food security and currently require massive pesticide applications to maintain crop yields. Many studies have demonstrated that rhizosphere microbiome composition and function, especially the interactions among microbes, and between plant and microbe are very important for soil‐borne disease suppression. By using SynComs, important progresses have been made in revealing the relationship between microbial interactions and resistance to pathogen invasion, and exploring the effects of rhizosphere microbiota‐plant interactions on disease suppression (Figure 1C).

Soil‐borne disease suppression is not only affected by the composition of rhizosphere microbial communities but is also determined by the interactions either between the resident microbial communities and the pathogens or within the resident microbial communities in the rhizosphere. A large number of studies which used inferred microbial co‐occurrence networks from community profiling or metagenomic data have suggested potential microbial associations (either positive, neutral, or negative) that may be linked with rhizosphere immunity. However, such correlative approach fails to reveal causal mechanisms through which rhizosphere microorganisms can affect each other, including soil‐borne pathogens. For such purposes, SynComs can be used to test interactions between resident populations under laboratory conditions, as well as strain–strain interactions that are important for plant resistance to pathogen infection. Moreover, Durán and colleagues combined metagenomics and SynComs approaches to show that negative interactions between prokaryotic and eukaryotic root microbiota members were critical for plant host resistance to pathogen and maintenance of host‐microbiota balance [9]. In conclusion, the combination of soil microbial community patterns used by omics‐based approaches with actual microbial interactions used of SynComs‐based approaches can better elucidate the relationship between microbial interactions and soil‐borne disease suppression.

There are numerous interactions between rhizosphere microorganisms and plant physiology, including immunity. Plant immune system directly participates in the defense against soil‐borne pathogens, it can also identify microbe‐associated molecular patterns using pattern‐recognition receptors, thus affecting the colonization of beneficial microorganisms in the rhizosphere. Although studies on plant‐rhizosphere beneficial microbial interactions have mainly focused on single‐model microorganisms, researchers recently began to pay attention to the relationship between SynComs and plant immunity. It is worth pointing out that Lebeis and colleagues first employed a SynCom composed of 38 different bacterial strains to explore the effect of plant immunity on the assembly of rhizosphere microbial communities. They found that plant immune signal‐salicylic acid modulated root colonization by specific bacterial families [10]. Castrillo et al. found that under P‐starvation, a SynCom composed of 35 bacterial strains enhanced the activities of P stress response genes PSR and PHR1, coordinating between nutrition and disease defense by rhizosphere microorganisms [11]. These studies suggest that the effects of the rhizosphere community on plant immunity present opportunities for the rational design of SynComs aimed at suppressing soil‐borne disease.

### SynComs applications for soil resilience

With soil resilience, we refer to the ability to preserve its functionality under stressors, multispecies communities may prevent substantial alterations in their average function against perturbations. In the soil environment, a single species is unlikely to perform multiple desired functions, the stabilizing effect of biodiversity may hence emerge as a consequence of functional redundancy when different species can perform a specific function but vary in their niche or sensitivity to environmental stressors. Therefore, soil biodiversity plays an important role in improving plant‐soil resilience to abiotic stresses. SynComs can help better explore the key interactions in diverse microbial communities driving resilience, For instance, microbe–microbe interactions, and plant–microbe interactions (Figure 1D).

Microbe–microbe interactions play important roles in almost all processes occurring on Earth, among which soil resilience to abiotic stresses [2]. Recently, fungi have been shown to facilitate colonization of dry soil by bacteria [12], and therefore studies investigating coinoculations of bacteria and fungi for improving soil resilience to drought are also of interest. Additionally, exopolysaccharides which are produced by an array of microorganisms like bacteria, cyanobacteria, microalgae, yeasts, and fungi impart defense against a wide range of environmental stresses. Such exopolysaccharides can facilitate microbe–microbe interactions, for instance quorum sensing, hence supporting plant growth in saline soils. Further, halophilic SynComs can remediate saline soils directly via improving nutrient status, soil structure, organic matter, pH, electrical conductivity, and deposition of ionic salts in soil [13].

Microbiomes play important roles in abiotic stress alleviation for plants by increasing water uptake and the bioavailability of nutrients, reducing oxidative stress, decreasing metal toxicity, producing plant hormones, and regulating various signaling pathways of plants. Nearly all abiotic stresses lead to the production of reactive oxygen species in plants, but some soil microorganisms which are associated with plants can release catalase, peroxidase, and other enzymes to reduce oxidative stress and hence reduce damage to the plant [14]. A SynCom combining five bacterial strains which were isolated from desert could promote tomato plant growth under saline and nonsterile conditions. This increased salt tolerance of tomato plants was associated with both expression of salt stress‐related genes and accumulation of iron in the shoots [15]. Additionally, exopolysaccharides produced by SynComs can facilitate microbe–plant interactions, and impart plant defense against environmental stresses [16].

## PRINCIPLES OF DESIGNING SYNCOMS TO ENHANCE SOIL HEALTH

Although we are technically able to apply SynComs in soil, there are still many challenges to overcome before we can precisely construct SynComs of designed function and efficacy that allow the translation of scientific findings to real‐world soil health improvements. For instance, SynComs application involves the deliberate release of microorganism in a treatment system to improve soil health, which often relates to an invasion process, that is, it requires the establishment of a SynCom, alien to a particular environment, in that environment while performing a desired functionality. Whether SynCom manages to successfully invade a microbial community depends on many factors, such as the structure and the interactions with and within the locally adapted resident community. Therefore, design and application of SynComs requires a deep mechanistic comprehension of all relevant interactions that take place during application into the resident microbiome, including the influence of soil physical and chemical properties and the interactions with resident plants and microbes in the soil.

**Figure 2:**
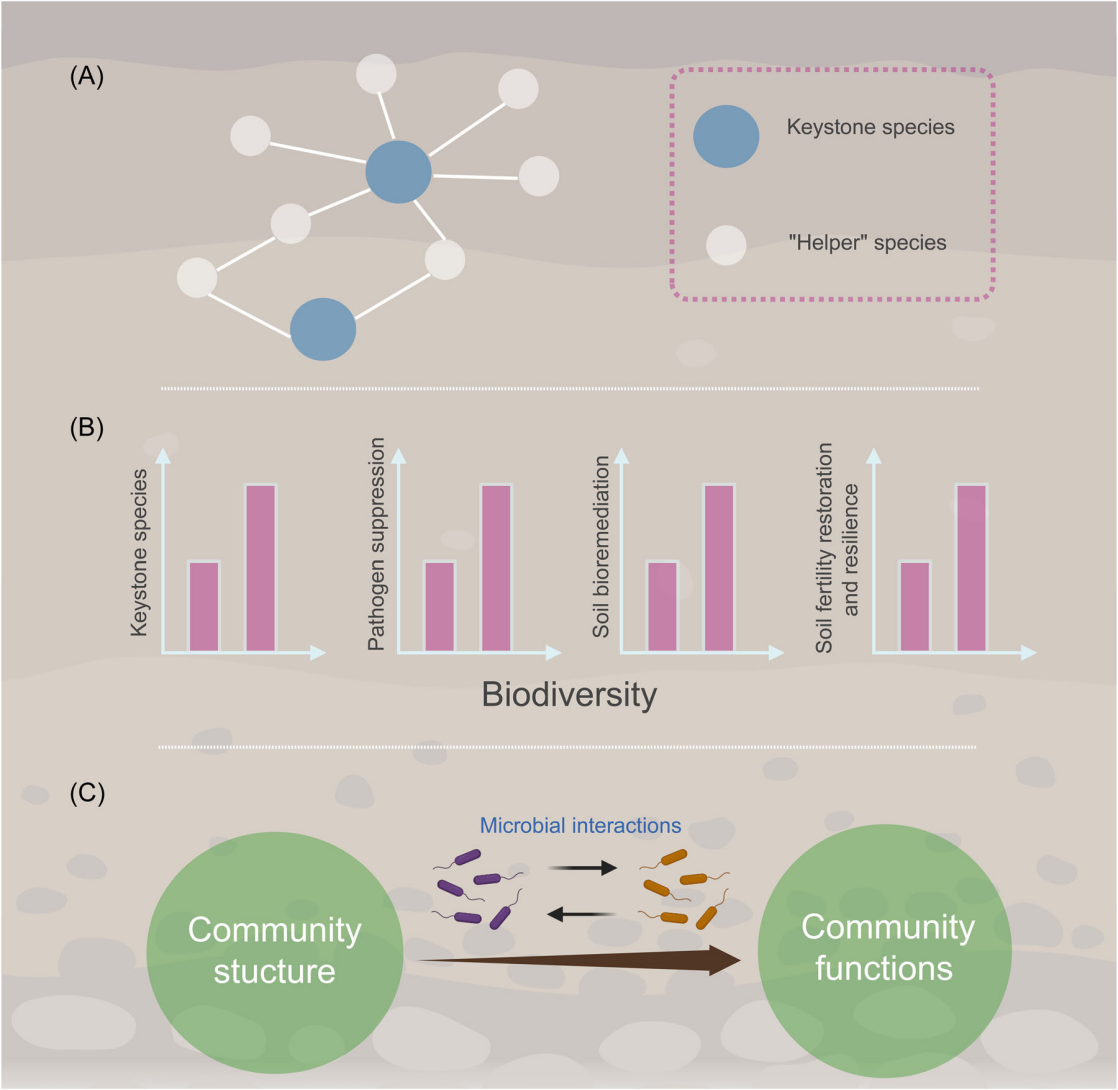
Principles for designing efficient SynComs to enhance soil health. (A) Keystone species and their “helper” species, (B) biodiversity, and (C) microbial interactions should be considered when designing SynComs to enhance soil health. The graphic was created with BioRender.com.

In the following sections, we summarize some ecological concepts that together form a useful set of guidelines to assess and understand the principle for designing efficient SynComs to enhance soil health (Figure 2).

### Keystone species and their “helper” species

A large number of studies have shown that core soil microorganisms play key roles in maintaining community functions. Like core microorganisms, we define keystone species as one or several microbial strains that are central in organizing community assembly and/or performing specific functions (such as pollutant degradation, key nutrient cycling, disease suppression, or soil resilience). For instance, related degrading strains need to be included in a SynCom for degrading the targeted pollutant, P‐solubilizing strains are necessary for designing a SynCom to increase phosphorus (P) bioavailability. By using a culture‐dependent method to track the abundance of each strain, Niu and colleagues investigated the role that each strain played during community assembly. They found the simple removal of *E. cloacae* led to the complete loss of the community function. This result suggests that *E. cloacae* plays the role of keynote species in this model ecosystem [17]. The species which can support these keystone species, such as promote their proliferation or colonize in a target environment, called their “helper” species. SynComs application which often involves a microbial invasion process will have a higher probability of success when resident “helpers” are present that either provide a niche for the invader or enhance the invader's fitness so that niches occupied by others are better colonized [7]. Therefore, including the keystone species together with their “helper” species when designing a SynCom improves its efficiency (Figure 2A).

### Biodiversity of SynComs

Species diversity affects most functions of microbial communities. A single strain is unlikely to perform multiple desired functions, and it is generally accepted that SynComs with higher richness have higher ability to enhance soil health (Figure 2B). SynComs with higher richness have more chance to include more keystone functional strains, hence providing more functions linked to soil nutrient bioavailability, soil resilience, plant nutrition, and hormonal balance than any single isolate. As there is usually more than one kind of pollutant in the soil, a SnyCom with higher diversity can contain strains that degrade different pollutants, thus achieving higher pollutant removal efficiency. Furthermore, because different species occupy different ecological niches and show different sensitivity to environmental pressures [18], SynComs with high richness can better survive and colonize complex environments such as contaminated soil. However, it should be noted that higher richness of a SynCom is not always beneficial. Under limited‐resource conditions, the increase of richness may aggravate the risk for resource competition among strains within the community, resulting in the decline of the overall biomass and the ability to enhance soil health.

### Microbial interactions

Interactions between the members of SynCom play an important role in its functions (Figure 2C). Positive interactions between strains at the same trophic level can result from public goods or metabolic cross‐feeding, where strains benefit from the presence of each other. Negative interactions may result from resource competition or direct interference competition where strains directly suppress each other via antagonism. These interactions may affect the function of SynComs in various ways. For pathogen invasion resistance, highly competitive resident communities are less prone to invasion if they can efficiently utilize and consume resources that would otherwise be available for pathogens. Furthermore, competing strains can inhibit each other directly by producing toxic metabolites, which can also inhibit the growth of pathogens, but facilitative communities may increase the number of resource niches by producing public goods that can also be utilized by invading pathogens. Conversely, cooperative community seems more efficient for soil bioremediation, improving soil resilience and fertility. For instance, contaminants in soil can be degraded or removed more efficiently by microbial synergy. Cooperation between bacterial strains with different growth‐promoting functions effectively improved N/P/K uptake and plant growth [5]. In addition to the interactions between strains at the same trophic level, the interactions between organisms at multiple trophic levels, such as predation and parasitism, should also be considered when designing efficient SynComs.

## FUTURE PERSPECTIVES

Although SynComs enable precise control over composition and manipulations such as strain dropouts and gene knockouts, the communities used are typically of low complexity (<20 strains), limiting their ability to model the biology of a native‐scale microbiome [19]. Therefore, there is a need to develop synthetic microbial communities with clear compositions and higher complexity to further investigate ecological principles. For instance, it should be possible to validate the positive or negative correlations within co‐occurrence networks with the actual strain interactions by designing more complex SynCom (e.g., >100 strains), assessing their specific metabolism using genome sequencing for each isolate to further reveal the intrinsic mechanisms of the core microbiome in enhancing soil health [20]. Additionally, it would be interesting in moving toward more realistic conditions, increasing environmental complexity, including biotic (such as complex communities containing bacteria, phages, fungi, and protozoa) or abiotic factors (such as soil pH, and nutrient availability) in studies on the interaction dynamics within microbial communities. Because screening microbial candidates is the crucial first step for SynCom design, one focus should be based on microbial functional properties to include strains with different specific functions in one SynCom. Further, to better understand the relationship between community structure and soil health, a reliable predictive model linking soil community structure and ecological function should be established, which combines ecological theory, basic experimental data, engineering development strategies (such as design‐build‐test‐learning cycle), and machine learning algorithms to design and apply SynComs.

## AUTHOR CONTRIBUTIONS

Zhong Wei provided direction and guidance throughout the preparation of this manuscript. Mei Li wrote the original draft. Mei Li and Jie Hu made the drawings in the manuscript. Mei Li, Jie Hu, Zhong Wei, Alexandre Jousset, Thomas Pommier, Xiangyang Yu, Yangchun Xu, and Qirong Shen reviewed and made significant revisions to the manuscript. All authors have read and approved the final manuscript.

## CONFLICT OF INTEREST STATEMENT

The authors declare no conflict of interest.

## Data Availability

This paper does not generate any new data. Supplementary materials (graphical abstract, slides, videos, Chinese translated version and updated materials) may be found in the online DOI or iMeta Science http://www.imeta.science/.
